# Increased associative interference under high cognitive load

**DOI:** 10.1038/s41598-022-05722-w

**Published:** 2022-02-02

**Authors:** Shira Baror, Moshe Bar

**Affiliations:** 1 The Gonda Multidisciplinary Brain Research Center, Bar Ilan University, 5290002 Ramat Gan, Israel; 2 Neuroscience Institute, New York University School of Medicine, New York, NY 10016 USA

**Keywords:** Human behaviour, Learning and memory, Visual system

## Abstract

Associative processing is central for human cognition, perception and memory. But while associations often facilitate performance, processing irrelevant associations can interfere with performance, for example when learning new information. The aim of this study was to explore whether associative interference is influenced by contextual factors such as resources availability. Experiments 1–3 show that associative interference increases under high cognitive load. This result generalized to both long-term and short-term memory associations, and to both explicitly learned as well as incidentally learned associations in the linguistic and pictorial domains. Experiment 4 further revealed that attention to associative information can delay one’s perceptual processing when lacking resources. Taken together, when resources diminish associative interference increases, and additionally, processing novel and ambiguous information is hindered. These findings bare relevance to other domains as well (e.g., social, educational), in which increased load or stress may prompt an undesirable bias towards prior, misleading information.

## Introduction

Associations are the building blocks of human cognition^1^. These are formed by the co-occurrence of events in our environment or in one-shot learning contexts and are utilized for the generation of predictions^2^ regarding likely upcoming events. Research shows that such associative-based predictions facilitate perception^3–5^, often in an implicit^6^ or unconscious^7^ manner. Nevertheless, while processing associations subserves many cognitive functions, associations can also interfere with one’s performance. In many domains, such as visual search^8^, learning a second language^9,10^, or avoiding stereotypical judgements^11,12^, rather than relying on stored associative information, optimally achieving one’s goals depends on the ability to exert selectivity, and keep previously acquired associations from biasing in the wrong direction. The central aim of this study was to examine whether associative interference depends on contextual factors, and specifically, whether lack of cognitive resources decreases or increases associative interference.

Although not focused on associative processing per se, much previous research has been devoted to understanding the relationship between resources availability and discarding irrelevant information, yielding conflicting results. Some studies propose that increased load facilitates selective attention, by reducing the capacity to process distracting information^13,14^. In line with these findings, it has even been proposed that using distraction as an emotion regulation strategy depends on having available resources to process the distracting information^15^. Others have shown that working memory capacity is necessary for selectively avoiding distraction, demonstrating that when resources diminish, increased interference, rather than a reduction in interference, takes place, hence suggesting that employing executive functions to discard the distracting stimulus requires resources^16,17^. To illuminate this discussion, here we probe the interaction between cognitive load and interference, and study this possible relation with regards to information carrying top-down associative information. In four experiments, we examine the influence of cognitive load on the processing of associations that are retained in short-term memory (STM) as well as in long-term memory (LTM; Exp. 1), associations that are incidentally learned in the linguistic (Exp. 2) and pictorial (Exp. 3) domains, and visual contextual associations (Exp. 4). The motivation for all four experiments was to test the influence of high cognitive load on activation of task-relevant associative information, as well as on the ability to discard task-irrelevant associative information. To preview the results, we found that while activation of associations is not compromised by high cognitive load, associative interference increases under high load.

## Results

### Experiment 1 (a & b)

**Figure 1:**
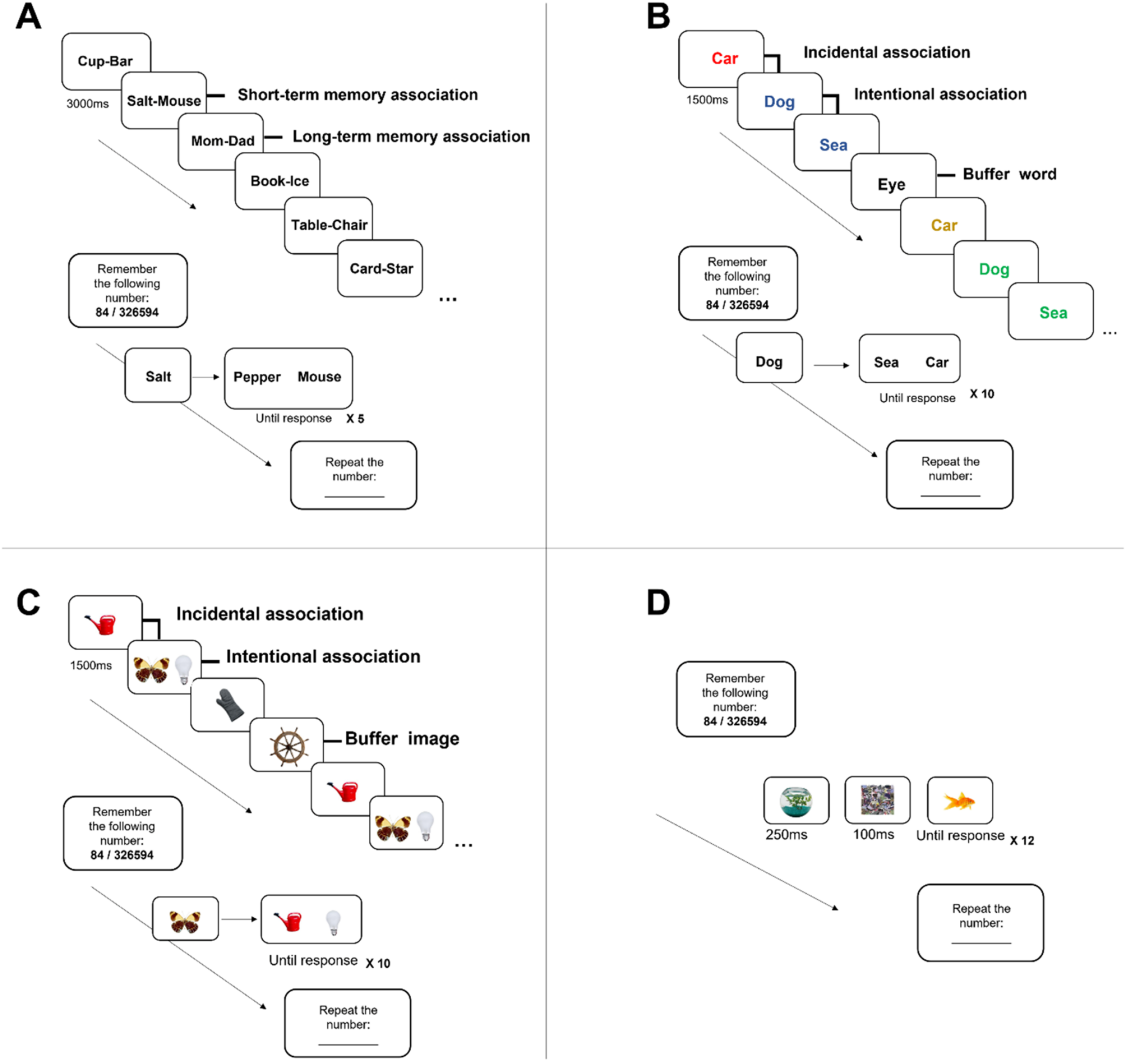
General experiments design. (**A**) Exp. 1. Learning: Participants were asked to associate words that appear together, which either formed a novel association or a LTM association. Every word pair was presented twice for 3000 ms. Test: Participants indicated which of two possibilities was the target associated with the cue. The distractor was either associated with the cue in LTM, or an unrelated buffer word. (**B**) Exp. 2. Learning: Participants were asked to associate consecutive words that appear in the same color. Every word pair was consistently preceded by the same word, to form an incidental association. Each stimulus was presented four times during learning, for 1500 ms. Test: Participants indicated which of two possibilities was the target associated with the cue. The distractor was either incidentally associated with the cue, or an unrelated buffer word. (**C**) Exp. 3. Learning: Participants were asked to associate images that appeared in pairs for a following memory test. Every image pair was consistently preceded by the same image, to form an incidental association. Each stimulus was presented four times during learning, for 1500 ms. Test: Participants indicated which of two possibilities was the target associated with the cue. The distractor was either the image incidentally associated with the cue, or an unrelated buffer image. (**D**) Exp. 4. Participants were asked to determine whether a target image appearing on screen is real or meaningless. In each trial, a prime appeared for 250 ms, followed by a 100 ms presentation of a mask. Following masking the image to be judged as object/non-object appeared for the maximum of 5 s or until response. In all experiments, test trials were run under high/low cognitive load condition. Participants were asked to remember 2 digits or 6 digits in the low and high load groups respectively. Presentation of the digit-string to remember, was followed by test trials of the main task, after which participants reported the digit-string they memorized. All images taken from Brady et al.^44^.

Exp. 1a examined how cognitive load influences processing of STM and LTM associations in the linguistic domain. Briefly, participants learned associations between word pairs, and subsequently, memory recognition of these pairs was tested under high or low cognitive load (using a digit-span task, see "General methods"). Learned associations were either novel, thus encoded in STM, or known, thus already stored in LTM. Recognition was carried using a two-alternative forced choice task (2AFC), in which participants were presented with a cue word that was learned with a paired word during the learning stage and were then asked to determine between two alternative words which was learned as an association with the cue word. Critically, the distractor in the recognition task could be either unrelated to the cue, or associated with the cue in LTM. This allowed us to examine three conditions of associative processing under high or low cognitive load: (1) recognition of a STM association in the face of an unrelated distractor; (2) recognition of a LTM association in the face of an unrelated distractor; (3) recognition of a STM association in the face of a distractor that is associated with the cue in LTM (see Fig. 1a and "Methods").

In analyzing the results, here and in all experiments reported henceforward, we first examined the differences between the load groups in accuracy performance in the cognitive load task, as a proxy for differences between the groups in resources availability. Note that reaction time (RT) measurement was not measured in the cognitive load task. Following the load-related accuracy analysis, we then conducted an accuracy and RT analyses of the main recognition task, probing the influence of cognitive load on associative activation and associative interference. Analysis of the main recognition task focused on trials in which the parallel cognitive load task was correctly performed. This strategy was aimed at controlling for the possibility that participants were not engaged in the cognitive load task when the digit strings were not properly remembered. An alternative strategy, in which performance in the main recognition task is examined independently from performance in the cognitive load task is presented in the Supplementary Materials.

Analysis of the cognitive load task revealed that subjects in the low load group (M = 0.93, SD = 0.11) were more successful than subjects in the high load group (M = 0.69, SD = 0.25) in remembering the digit strings (t(44) = 4.23, *p* < 0.0009, Cohen’s d = 1.24).

Next, we assessed performance in the main recognition task. We conducted repeated measures RT and accuracy ANOVA, with load as a between-subjects variable, and memory recognition conditions as a within-subjects variable. RT analysis, which focused on correct responses only, did not show significant differences between participants in the low load group (M = 1212 ms, SD = 160 ms) and participants in the high load group (M = 1226 ms, SD = 183 ms; *p* < 0.79). Nonetheless, significant RT differences were found between the memory recognition conditions, such that RT was significantly faster in the condition in which the target was associated with the cue in LTM and the distractor was unrelated to the cue (F(43,2) = 40, *p* < 0.0009, η2*p* = 0.65).

**Figure 2:**
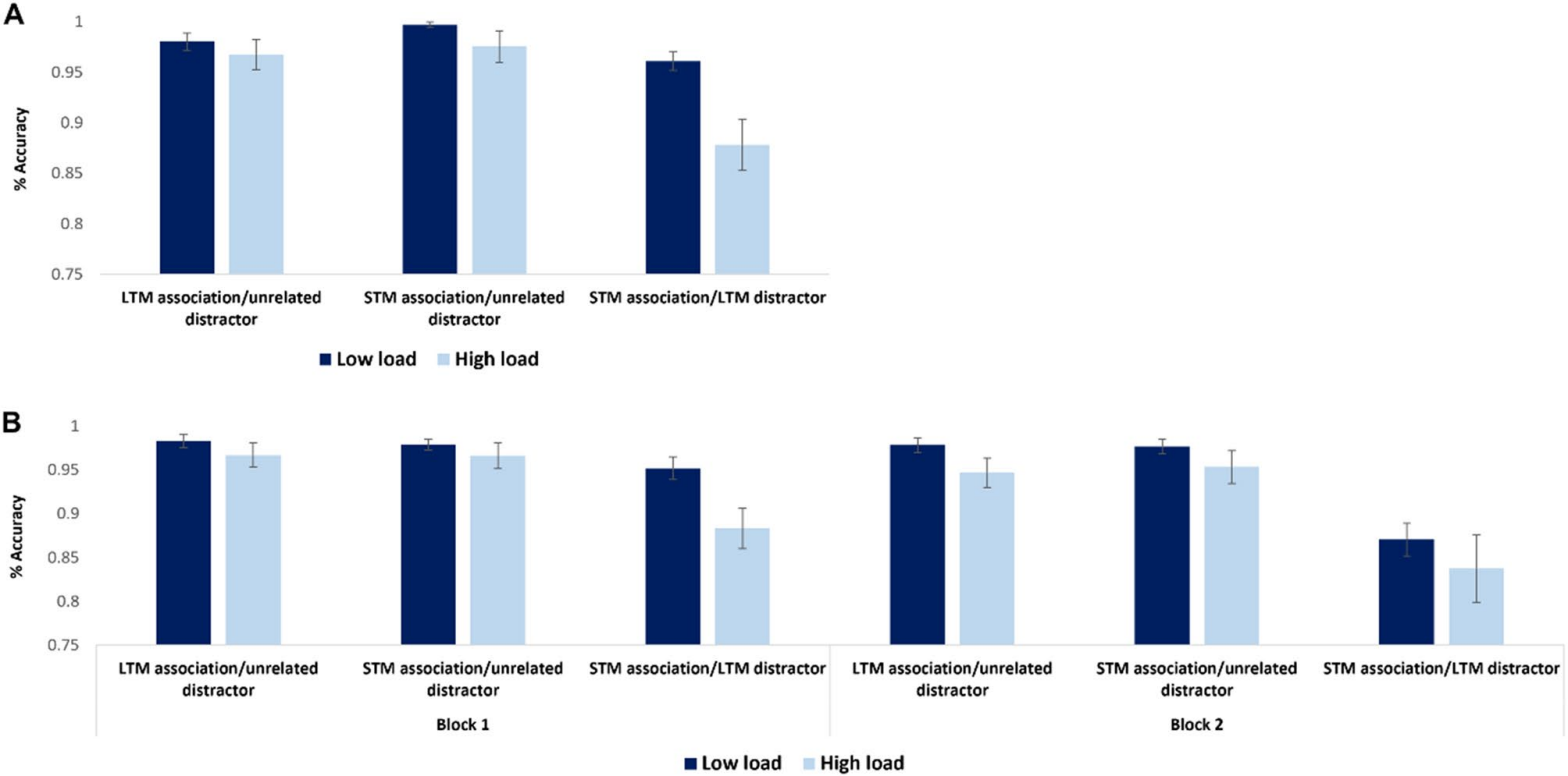
Results in Exp. 1a (**A**) and Exp. 1b (**B**). In each condition, left = target; right = distractor. (**A**) Main effect of load: *p* < .01, Main effect of recognition condition: *p* < .0009. Load X recognition condition Interaction: *p* < .009. (**B**) Main effect of load: *p* < .03, Main effect of block: *p* < .01, Main effect of recognition condition: *p* < .0009. Block X recognition condition Interaction: *p* < .007. Error bars indicate standard error of the mean.

Accuracy analysis of the main recognition task showed a significant effect of load. Recognition accuracy was lower under high load (M = 0.94, SD = 0.01) compared with under low load (M = 0.98, SD = 0.01; t(44) = 6.9, *p* < 0.01, η2*p* = 0.13). Additionally, memory recognition conditions were significantly different from one another, such that recognition was significantly compromised when the distractor was associated in LTM with the cue (F(43,2) = 11.45, *p* < 0.0009, η2*p* = 0.34). Critically, an interaction emerged between load and memory recognition conditions (F(43,2) = 5.03, *p* < 0.009, η2*p* = 0.1). Subsequent pairwise comparisons revealed that participants under high load were significantly worse than participants under low load in recognizing STM associations when the distractor was associated in LTM with the cue (t(44) = 3.11, *p* < 0.003, Cohen’s d = 0.9 ; Fig. 2a).

Together these findings suggest that high cognitive load does not diminish activation of either STM or LTM associations, but rather limits the ability to discard irrelevant LTM associations, leading to increased associative interference.

The accuracy and RT analyses reported here included only trials in which performance in the cognitive load task was correct. However, since success rate in the cognitive load task was significantly compromised under high load, the inherent imbalance between the number of trials included in the high load and the low load groups may have biased our results. To control for this imbalance, two additional sets of analyses were conducted (in this experiment, as well as in all four experiments reported in the study). The first, allowed a softer inclusion criterion, such that under high load, trials with a minor error in the parallel cognitive load task were included (one digit omission or one switch, out of the 6 digits to remember). The second set of analyses included all trials of the main task, regardless of success in the cognitive load task. These analyses minimize the imbalance between the number of trials analyzed in each load group and show similar results to the ones reported here (see Supplementary A for a full comparison between these analysis sets in all experiments).

To examine whether the findings from Exp. 1a extend to other forms of load, Exp 1b was conducted. This experiment was identical to Exp. 1a, with the only difference that the availability of resources was manipulated both by cognitive load, as well as by extending the experiment, so that it comprised two full blocks of the task (see "Methods"). Hence, beyond the between-subjects cognitive load manipulation, a within-subjects manipulation was incorporated such that performance in the first block was compared to performance in the second block. It was hypothesized that as the experiment extends, internal resources for completing the task will diminish. If this was true, it was expected that independent from the level of cognitive load imposed, associative interference will increase in the second block compared with the first block.

As in Exp. 1a, analysis began with examining performance in the cognitive load task. This analysis revealed that participants in the low load group (M = 0.94, SD = 0.07) were more successful in remembering the digit strings compared with participants in the high load group (M = 0.67, SD = 0.2; t(42) = 5.18, *p* < 0.000007, Cohen’s d = 1.6).

Turning to analyses of the main recognition task, we conducted repeated measures RT & accuracy ANOVA, with load as a between-subjects variable. Memory recognition conditions as well as time into the experiment (block 1 vs. block 2) were the within-subjects variables. RT analysis did not reveal significant differences between participants in the low load group (M = 1115 ms, SD = 184 ms) and participants in the high load group (M = 1185 ms, SD = 150 ms, *p* < 0.11). No RT effect was found for blocks as well (block 1: M = 1158 ms, SD = 174 ms; block 2: M = 1141 ms, SD = 154 ms, *p* < 0.34). Nevertheless, a main effect was found for memory recognition conditions, such that RT was significantly faster when the target formed a LTM association with the cue and the distractor was unrelated (F(41,2) = 38.79, *p* < 0.0009, η2*p* = 0.65).

Accuracy analysis revealed three main effects. First, a main affect of load was found. Lower recognition accuracy levels were found for participants under high load (M = 0.92, SD = 0.01) compared with participants under low load (M = 0.95, SD = 0.01; t(42) = 4.57, *p* < 0.03, η2*p* = 0.09). Second, a main effect was found for the memory recognition conditions. Lowest accuracy rates were found when the target formed a STM association with the cue, and the distractor formed a LTM association with the cue (F(41,2) = 25.66, *p* < 0.0009, η2*p* = 0.44). Thirdly, a main effect was found for time into the experiment, such that higher recognition accuracy was measured in the first block (M = 0.95, SD = 0.007) compared with the second block (M = 0.92, SD = 0.01; F(42,1) = 6.44, *p* < 0.01, η2*p* = 0.13).

Importantly, an interaction emerged between blocks and memory recognition conditions (F(41,2) = 5.21, *p* < 0.007, η2*p* = 0.15). Subsequent pairwise comparisons revealed that extending the experiment to include two blocks rather than one, selectively led to greater interference by the distractor that formed a LTM association with the cue (t(42) = 3.02, *p* < 0.004, Cohen’s d = 0.45; Fig. 2b). This finding further supports the hypothesis that resources are specifically required for exserting selectivity when associations interfere with task goals.

Exp. 1a and 1b focused on explicitly learned associations and showed that while activation of STM and LTM associations is not compromised under high cognitive load, irrelevant LTM associations pose greater interference on task performance when available resources are limited. The next experiment examined whether these findings are generalizable to incidentally learned associations as well.

### Experiment 2

In Exp. 2, participants intentionally learned novel associations between two consecutively presented words, while additional associations were introduced via incidental learning (see Fig. 1b and "Methods"). Memory recognition of intentionally learned associations was tested in a 2AFC task under varying cognitive load conditions. Here, during the recognition task, the distractor could have either been completely unrelated to the cue, or alternatively, associated with the cue via incidental learning. It was hypothesized that if the findings from Exp. 1 generalize to incidentally learned associations, then increased associative interference by irrelevant incidental associations will be observed under high cognitive load.

First, performance in the cognitive load task was examined, revealing that participants in the high load and low load groups were significantly different from one another in performing the digit-span task (low load: M = 0.94, SD = 0.08; high load: M = 0.61, SD = 0.28; t(41) = 5.26, *p* < 0.0009, Cohen’s d = 0.8).

Turning to the main recognition task, we conducted repeated measures RT & accuracy ANOVA, with load as a between-subjects variable, and memory recognition conditions as a within-subjects variable. RT analysis did not show significant RT differences between participants in the low load group (M = 1449 ms, SD = 210 ms) and participants in the high load group (M = 1612 ms, SD = 397 ms, *p* < 0.1). No RT effect was found for the memory recognition conditions as well (unrelated distractor: M = 1544, SD = 329 ms; incidental distractor: M = 1517, SD = 358 ms, *p* < 0.47).

**Figure 3:**
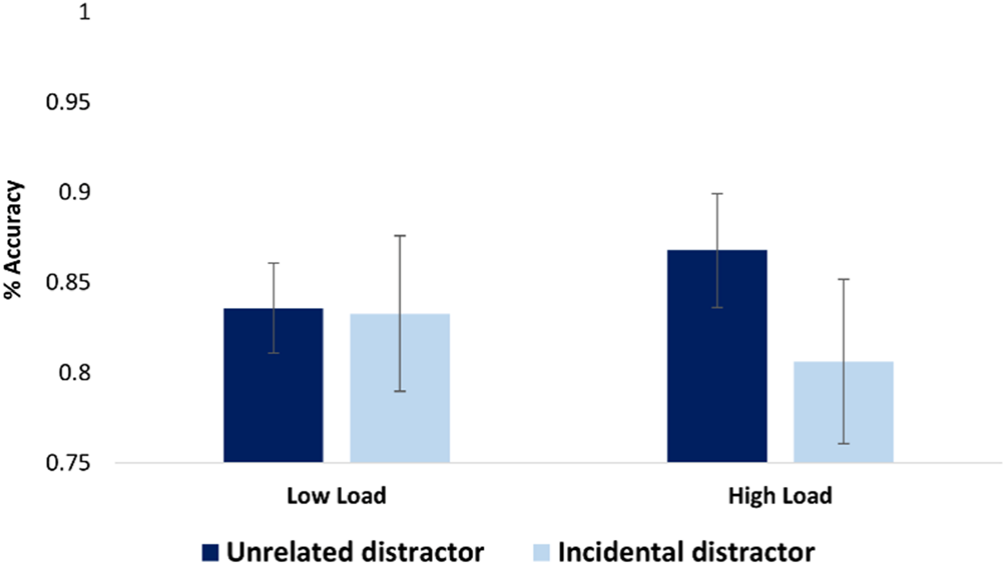
Results in Exp. 2. Main effect of recognition condition: *p* < .02. Load X recognition condition interaction: *p* < .03. Error bars indicate standard error of the mean.

Accuracy analysis did not show significant differences between participants in the low load group (M = 0.83, SD = 0.13) and participants in the high load group (M = 0.83, SD = 0.12, *p* < 0.9). Nevertheless, analysis showed a significant main effect for memory recognition conditions, revealing that independent from load, better performance was found when the distractor was unrelated to the cue (M = 0.85, SD = 0.11) compared with when the distractor was incidentally associated with the cue (M = 0.82, SD = 0.14; F(41,1) = 5.7, *p* < 0.02, *η2p* = 0.12). Critically, an interaction emerged between load group and memory recognition conditions (F(40,1) = 4.64, *p* < 0.03, η2*p* = 0.1; Fig. 3). A subsequent pairwise comparison revealed that in the high load group, participants performed significantly worse when the distractor was incidentally associated with the cue compared with when the distractor was not associated with the cue (F(20,1) = 7.56, *p* < 0.01, Cohen’s d = 0.42), pointing to increased associative interference under high cognitive load.

To further examine whether these results generalize beyond the linguistic domain, we conducted Exp. 3, which involved pictorial rather than word-based associations.

### Experiment 3

Exp. 3 was similar in its design to Exp. 2 with the difference that it involved image pairs rather than word pairs as stimuli (see Fig. 1c and "Methods"). It was hypothesized that similarly to Exp. 2, increased associative interference by incidentally learned associations will be found under high cognitive load.

First, analysis of performance in the cognitive load task showed that participants under low load were significantly better in the cognitive load task compared with participants under high load (low load: M = 0.9, SD = 0.11; high load: M = 0.59, SD = 0.24; t(48) = 5.26, *p* < 0.0009, Cohen’s d = 1.59).

Next, to analyze performance in the main recognition task, repeated measures RT & accuracy ANOVA were conducted, with load as a between-subjects variable, and memory recognition conditions as a within-subjects variable. RT analysis did not yield significant differences between the low load group (M = 1250 ms, SD = 354 ms) and the high load group (M = 1228 ms, SD = 296 ms, *p* < 0.8). No RT effect was found for the memory recognition conditions as well (unrelated distractor: M = 1258, SD = 320 ms; incidental distractor: M = 1236, SD = 327 ms, *p* < 0.14).

**Figure 4:**
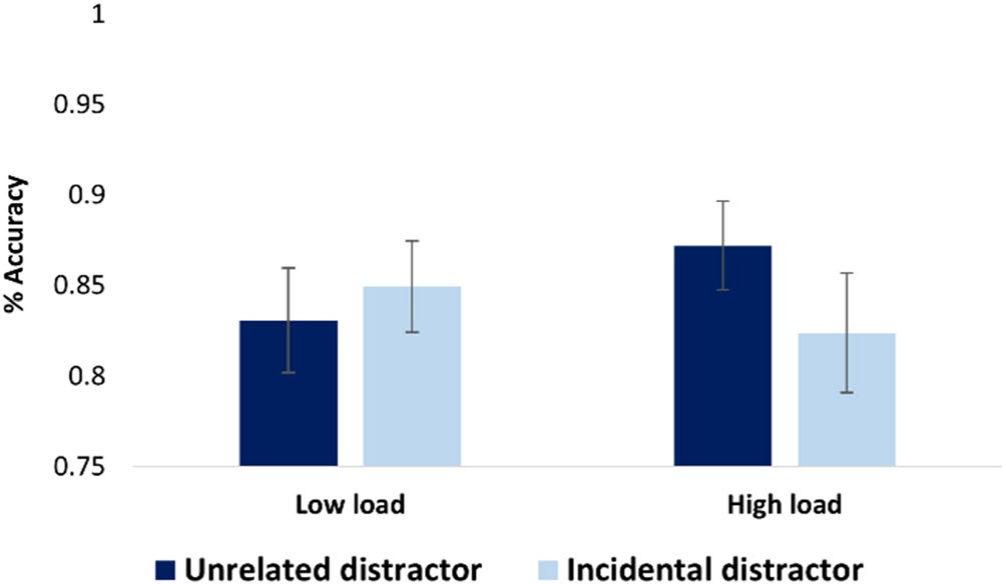
Results in Exp. 3. Load X recognition condition interaction: *p* < .009. Error bars indicate standard error of the mean.

Accuracy analysis did not reveal a main effect of load (low load: M = 0.84, SD = 0.13; high load: M = 0.84, SD = 0.14, *p* < 0.8) nor did it reveal a main effect for memory recognition conditions (unrelated distractor: M = 0.85, SD = 0.13; incidental distractor: M = 0.83, SD = 0.14, *p* < 0.18). Nevertheless, the analysis revealed an interaction between load and memory recognition conditions (F(48,1) = 4.64, *p* < 0.009, η2*p* = 0.15). Subsequent pairwise comparison revealed that under high load, subjects performed significantly worse when the distractor was incidentally associated with the cue compared with when the distractor was unrelated to the cue (t(25) = 2.76, *p* < 0.01, Cohen’s d = 0.58). Extending the findings from Exp. 2, this result points to increased associative interference by incidentally learned associations in the pictorial domain as well (Fig. 4).

Taking Exp. 1, 2 and 3 together, the results demonstrate that in a memory recognition context, under high cognitive load associative activation is not diminished, and associative interference increases.

To further examine the influence of cognitive load on associative processes at a finer timescale, Exp. 4 was conducted. This experiment utilized visual contextual associations in a priming paradigm, allowing to test whether cognitive load influences the processing of contextual associations in a rapid, perceptual processing context.

### Experiment 4

Exp. 4 employed a visual contextual priming paradigm. Prime-target pairs appeared consecutively, and participants were asked to make an object/non-object decision regarding the target. As in the previous experiments, a cognitive load task was conducted in parallel (see Fig. 1d and "Methods"). Targets were either a real object, or an abstract sculpture (i.e., a non-object). Among the targets of real objects, 50% of the targets were associated to the prime in LTM, and 50% were unrelated to the prime image. Using this rapid contextual priming paradigm was aimed to probe associative processing at finer timescales compared with the relatively slow RT measured in the previous experiments. It should be notes that as this experiment relies solely on LTM associations, a learning stage was not employed. Nevertheless, here we further controlled for the level of associativity between the prime and the target images, by conducting a pilot study in which three levels of prime-target contextual associativity were established, namely: strong, intermediate, and weak (see Supplementary B). Briefly, relying on the pilot-participants’ responses, strong associativity pertained to primes that elicited few, specific associations that belong to one context (e.g., a dog’s bone elicited the association of a dog); intermediate associativity pertained to primes that elicited many associations that belong to the same context (e.g., a grocery shopping bag elicited many associations in the context of grocery shopping, such as: milk, vegetables, bread, etc.); and weak associativity pertained to primes that elicited many associations that belong to many different contexts (e.g., binoculars elicited many associations from different contexts, such as bird watching, army, or hiking).

As in the previous experiments, performance in the cognitive load task was first analyzed, revealing that participants in the low load group (M = 0.91, SD = 0.01) performed better than participants in the high load group (M = 0.83, SD = 0.01; t(48) = 3.01, *p* < 0.004, Cohen’s d = 0.85).

**Figure 5:**
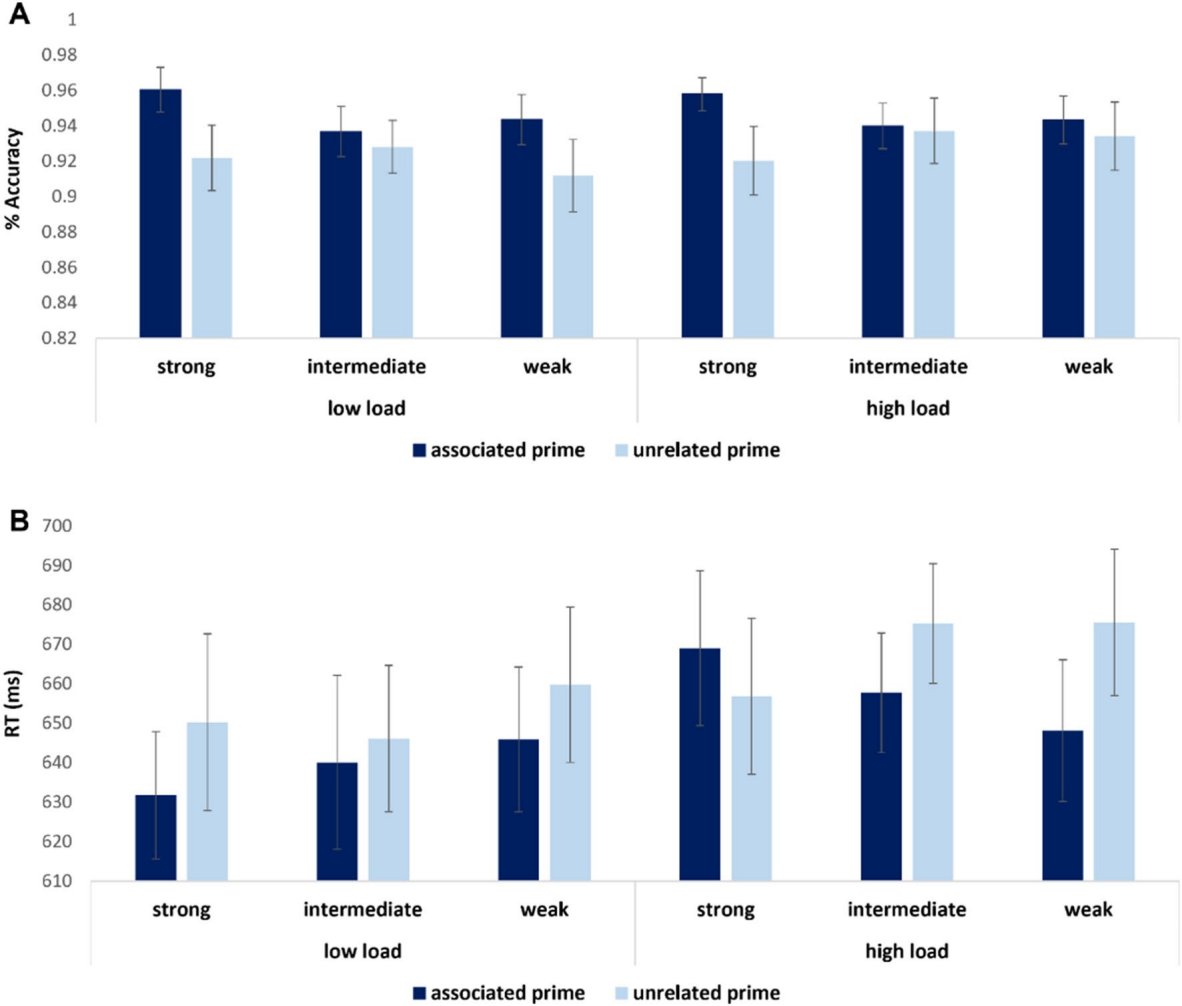
Results in Exp. 4: (**A**) Accuracy. Images that were primed with an associated image were judged more accurately as real objects compared with images following an unrelated prime (*p* < .004). (**B**) RT. Images that were primed with an associated image were judged faster as real objects compared with images following an unrelated prime (*p* < .01). Error bars indicate standard error of the mean.

Next, we analyzed performance in the object/non object task. To that aim, we conducted repeated measures RT and accuracy ANOVA, with load as a between-subjects variable, and associativity (strong/intermediate/weak) and priming (primed/unrelated) as within-subjects variables. Note that these analyses included only trials in which a real object was presented as target. Accuracy analysis did not yield significant differences in performance between the low load group (M = 0.93, SD = 0.07) and the high load group (M = 0.94, SD = 0.07, *p* < 0.64**)**. Nevertheless a significant main priming effect was revealed such that accurate recognition of targets as real objects was facilitated when targets were preceded by associated primes (M = 0.94, SD = 0.008) compared with when targets were preceded by unrelated primes (M = 0.92 SD = 0.1; F(48,1) = 9.26, *p* < 0.004, η2*p* = 0.16; Fig. 5a). Next, similarly to the accuracy analysis, RT analysis did not yield significant differences between the low load group (M = 645 ms, SD = 97 ms) and the high load group (M = 677 ms, SD = 103 ms, *p* < 0.23**)**. However, this analysis revealed a significant priming effect. Participants were significantly faster in recognizing objects that were preceded by an associated prime (M = 648 ms, SD = 11 ms) compared with objects that were preceded by an unrelated prime (M = 660 ms, SD = 13 ms; F(48,1) = 5.96, *p* < 0.01, η2*p* = 0.11, Fig. 5b).

Importantly, a triple interaction was found between priming, associativity strength and load conditions (F(47,2) = 3.74, *p* < 0.03, η2*p* = 0.12).

Conducting subsequent analysis revealed that when target objects were primed with an associated cue, the prime-target association’s strength interacted with load group. Under low load, the stronger the prime-target association, the faster object recognition took place. In contrast, under high load an opposite pattern emerged, such that stronger prime-target associations delayed task performance (F(48,2) = 3.8, *p* < 0.02, η2*p* = 0.09; Fig. 5b, dark columns).

Taken together with the findings in the accuracy analyses, these results imply that while judging that an image depicts a real object was facilitated by strong contextual associations in both load conditions, this accuracy enhancement was accompanied with a delayed RT in the high load condition. This finding may attest to increased attention to contextual associative information when resources diminish.

#### Exploratory analysis of non-objects

Analysis of task-performance in Exp. 4 capitalized thus far on recognition of real objects in the object/non object task. Nevertheless, by design, 50% of the targets presented in Exp. 4 were non-objects, that similarly to real objects, were preceded by primes that elicit strong, intermediate or weak associations. Here, an exploratory analysis was conducted to expand our investigation and examine whether contextual associations that may have been triggered by the primes influenced the perception of non-objects as well. Although non-object recognition processes may differ qualitatively from object recognition processes, it was reasoned that the facilitation effect found for real objects that are associated with the primes, may be mirrored in delayed recognition of non-objects when these associations were disproved. To examine this hypothesis, we tested how judgements of non-objects were influenced by the associativity level of the primes in each load group. To this aim, the non-object targets were divided into three groups, according to the priming image that preceded them, i.e., non-objects that followed images that elicit strong, intermediate or weak associations. We then conducted repeated measures ANOVA for accuracy and RT, with load as a between subject variable, and associativity of the prime as a within-subject variable.

**Figure 6:**
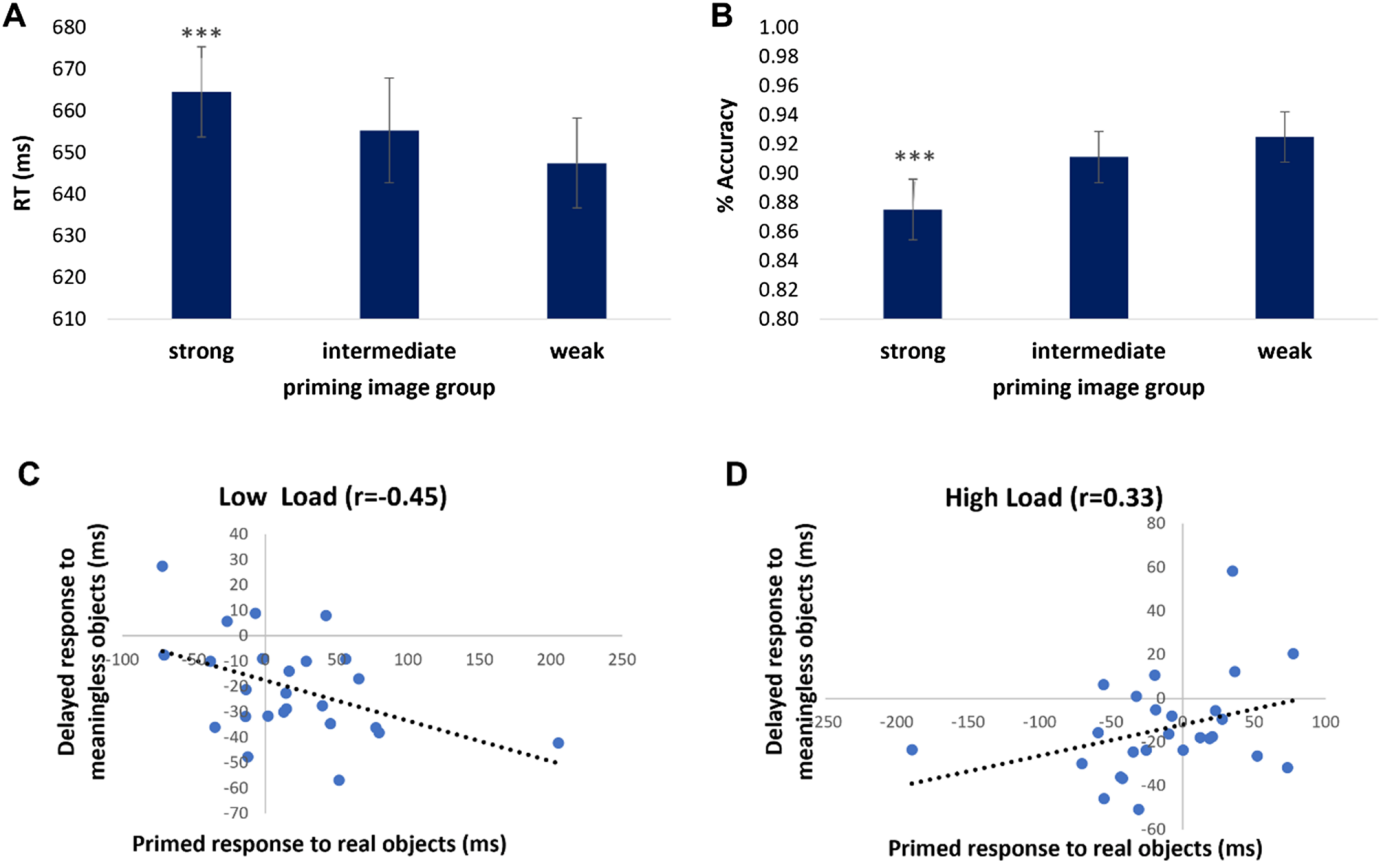
Negative priming of meaningless objects. (**A** + **B**) Judgements of meaningless objects were influenced by the prime image that preceded them. The stronger the associative information the prime triggered, the slower the response to meaningless objects (*p* < .0009) as well as the less accurate (*p* < .0009). (**C** + **D**) In each load group, participants’ enhanced responses to real objects that were primed with a prime from the strong associativity condition (measured as RT(images unrelated to strong primes)—RT (images associated to strong primes)) correlated with delayed responses to meaningless objects that were primed with the strong prime associativity condition (measured as RT(weak primes)—RT (strong primes)). This correlation was significant in the low load group (*p* < .02) and showed a similar yet opposite trend in the high-load group (*p* < .09). Error bars indicate standard error of the mean.

Accuracy analysis did not reveal a main effect for load group (low load: M = 0.88, SD = 0.16; high load: M = 0.91, SD = 0.1, *p* < 0.52), yet revealed a significant effect for the primes’ degree of associativity. Non-objects were judged significantly less accurately when they appeared following primes that elicit strong associations (M = 0.87, SD = 0.02) compared with non-objects appearing following primes that elicit intermediate or weak associations (M = 0.91, SD = 0.18 and M = 0.92, SD = 0.17 respectively; F(48,2) = 13.28, *p* < 0.0009, η2*p* = 0.37, Fig. 6a). RT analysis revealed a similar pattern of results. No effect was found for load group (low load: M = 657 ms, SD = 90 ms; high load: M = 672 ms, SD = 81 ms, *p* < 0.53) yet a significant effect was found for the primes’ degree of associativity, such that non-objects were judged significantly slower following primes that elicit strong associations (F(48,2) = 16.11, *p* < 0.0009, η2*p* = 0.38, Fig. 6b).

Lastly, we examined whether the influence of primes that elicit strong associations on real-object recognition correlated with the influence of those primes on non-object recognition. Exploring this hypothesis, a Pearson correlation was computed between the priming of real objects (measured by RT differences between responding to primed and unprimed real objects in the strong association prime group) and the delayed response to non-objects (measured by RT differences between recognition of non-objects that followed primes eliciting strong associations and recognition of non-objects that followed primes eliciting weak associations). This correlation analysis was done separately for each load group.

In the low load group, analysis revealed a significant and negative correlation (r = − 0.45, *p* < 0.02). The enhancement in object recognition following primes that elicit strong contextual associations, correlated with the delay in recognizing non-objects following similar primes (Fig. 6c). In the high load group, analysis revealed a trend towards an opposite, positive correlation between delayed object recognition and delayed non-object recognition. This correlation however did not reach significance (r = 0.33, *p* < 0.09, Fig. 6d). This exploratory analysis opens new questions regarding the possible ‘price’ of relying on associations when attending to meaningless and ambiguous information. It has been suggested in the past that stimulus ambiguity is associated with increased top-down involvement^18^, so it is possible that when presented with ambiguous information here, participants were influenced to a great extent by strong top-down associations elicited by the primes.

## Discussion

The current study examined whether associative interference increases, or alternatively decreases, when available resources diminish. The findings show that under high cognitive load, associative activation is maintained, and associative interference increases, suggesting that high cognitive load disrupts the selective discounting of irrelevant associative information. Bridging efficient associative processing with minimizing associative interference when needed, these findings point to a possible two-components mechanism: an initial resources-free process of associative activation, followed by a selective, resources-consuming process of discarding those associations that are unhelpful.

The first three experiments focused on memory performance, and showed that resources depletion uniquely affected memory performance when the distractor was associated with the cue, primarily interfering with attenuating the influence of irrelevant associations, rather than with activating relevant associations in memory. Real-life situations involve activation of various stored associations, so minimizing associative interference is crucial for coherent thought and here seems to require available resources. As such, our findings conform to research showing that memory deficits in older adults result from increased interference of associated yet irrelevant information, rather than from lack of memory activation^19,20^. In that context, it would be of relevance to examine age-related changes in associative interference.

Furthermore, our findings regarding interference by prior associations corroborate and extend previous discussions regarding how load influences discarding irrelevant cues in other domains, such as perception. When engaging with the external environment, perceptual cues of low-level saliency (e.g., color) capture attention in what some suggest is an automatic manner^21,22^. While this encompasses survival benefits in rapidly avoiding danger, an observer must constantly attenuate attentional capture to avoid distraction. Some studies suggested that this attenuation is carried through resources-consuming inhibition^23^. The findings presented here suggest that associative information, whether learned explicitly or implicitly, encompasses internal saliency, and captures attention that needs to be maneuvered, and perhaps inhibited, similarly to an exceptionally bright or large object in the external surroundings. Important in this context is ‘load theory’^24,25^ which suggests that applying high perceptual load exhausts one’s capacity to process perceptually irrelevant information, resulting in attenuation of distractor-based interference. This theory also suggests that under low perceptual load, selective attenuation of irrelevant information still depends on the availability of cognitive control resources. As our tasks did not involve high perceptual load, but rather a cognitive form of load, our findings are in line with the load theory, showing that cognitive resources are needed to avoid distraction, and extend this line of work to the associative processing domain, which, as suggested above, introduces a top-down form of distraction, rather than a distraction based on perceptual saliency. In this context, whether perceptual load would attenuate interference by top-down associative processing remains an open question. Some supporting evidence to this direction is found in Forester and Lavi^26^ who found that perceptual load minimizes the phenomenon of task-unrelated thought, a phenomenon that can be interpreted as distraction by irrelevant associative processes. The findings in Forester and Lavi^26^ were obtained in the context of a visual-search task, so it would be of importance to examine whether associative interference is attenuated by perceptual load when the task at hand relies on one’s stored knowledge rather than on their perceptual experience.

The findings in the fourth experiment show that primes that elicit strong associations enhance correct recognition of subsequent objects, but that while this enhancement is accompanied with accelerated responses under low load conditions, under high load conditions it is accompanied with delayed responses. This finding implies that under high load, memory-guided attention may slow down performance. This relates to two previous accounts which were proposed for object recognition and perceptual processing, and which incorporate a selective stage in addition to a rapid, associative activation stage. The first account proposed that object recognition involves an object-based triggering of associations from multiple context frames, and a context-based process that narrows these ‘initial guesses’ to the most contextually appropriate ones^27^. The second account, using a repetition priming paradigm^28^, suggested that efficient fine-tuning of the visual representation that is caused by repetition, is followed by a selective process in which only key features of the input remain represented. With relation to both accounts, our findings seem to indicate that taxing resources interferes primarily with the selective process, leaving the stage of associative activation intact. Future studies are required to delineate how resources limitations influence associative processing at different brain regions and at different time points of perception and recognition. For example, previous work using fMRI methods, suggests that there exists a balance between hippocampal activity that relates to processing novel stimuli, and prefrontal activity that is engaged in monitoring stored, potentially interfering information^29^. Additionally, with regards to temporal dynamics, some work done by means of EEG suggests that low frequency phase synchronization is predictive of working memory load^30^. Considering our findings, combining these methods in the future may possibly allow pinpointing the spatio-temporal mechanisms through which load influences associative activation and associative interference.

The exploratory analysis in Exp. 4 showed that non-object recognition is significantly hindered when it follows the presentation of a strongly contextualized prime. This finding corresponds with evidence from false memory studies showing that strong associativity between events^31^ and strong ‘gist’^32,33^ can lead people to falsely ‘recall’ seeing related events that never really happened. Our findings extend this line of work by implying that the more one relies on learned associations, the less one is likely to discern information propagating in bottom-up routes, elucidating the potential cost in relying on prior knowledge when perceiving novel information, such as when observing art^34^, or when encountering atypical behavior^35^.

A recent theoretical framework that speaks to this tension between relying on top-down priors and bottom-up inputs is the Overarching States of Mind framework^36^. Building upon literature that characterizes behavior along the exploration–exploitation axis^37^, this framework suggests that in a state of exploitation, processing top-down associative information is enhanced, influencing cognition to a greater extent than in the state of exploration. It is possible that high cognitive load shifts people towards exploitation, thus increasing reliance on prior knowledge, despite contravening one’s goals. Our findings imply that while sustaining a state of exploration will not reduce the automatic activation of irrelevant associations, it may nevertheless attenuate their interference.

Finally, our findings bare relevance to other frameworks of information processing. For example, theories of attitude formation suggest that attitudes involve both activation in the associative structure of stored information as well as propositional processes of reasoning which relate to evaluating that information’s credibility and relevance^38^. It is possible that high load, or more generally a state of exploitation, attenuates propositional processing, thus diminishing the ability to critically evaluate and ultimately filter prior misleading information. This account may underlie previous findings showing that high load increases reliance on false information^39^ and findings showing that threat, which is interpreted as a form of load, leads to greater anchoring towards prior decisions^40^. We propose that other realms such as marketing, communication, education or even law investigations, should be informed that high load, like stress and pressure, may prompt comprehensive memory-related, perceptual, and behavioral biases towards stored associative information.

## Concluding remarks

Years of research have shown that associative processing is involved in numerous cognitive and perceptual facets of the human experience. Our results extend previous findings in two main directions. First, we show that people are more prone to be misguided by prior associations under high cognitive load. Second, relying on top-down associations directly results in decreased processing of bottom-up input. These key points contribute to understanding the conditions in which relying on associations can be utilized in favor of one’s goals. We conclude that when limited in resources, relying on associative information may result in cognitive, and perhaps social and emotional costs.

## Methods

### General methods

All experiments were approved by the Bar-Ilan University, Gonda Brain Research Center Ethics Committee, and were performed in accordance with the guidelines and regulations. All participants signed an informed consent form prior to participation and received course credit or monetary compensation for their participation. All participants were native Hebrew speakers, with normal or corrected to normal vision, and were screened for attention and other neurological deficits.

All experiments involved a primary task that was aimed to trigger associative processing (see specific experiments below), and a secondary, cognitive load task (Fig. 1). The cognitive load task employed an adaptation of the digit span task, which has been shown to rely on working memory resources^41^, and as was done in previous studies^42^. Subjects were requested to hold in mind strings of two or six digits, in the low load and high load groups respectively and were later asked to repeat them explicitly by typing them into a textbox. Digit strings were chosen randomly, alternating in every experimental set. In all experiments, the primary task (memory/object recognition task) was manipulated within subjects, and the cognitive load task was manipulated between subjects.

The general procedure was identical across experiments. After signing an informed consent form, participants were randomly assigned to either the low or the high load group, unbeknownst to the experimenter. Subsequently, participants were seated in a quiet room, ~ 60 sm away from an LCD monitor with a resolution of 1920 × 1200 pixels and a refresh rate of 60 Hz, on which the experimental stimuli were presented. After being given instructions, participants took part in the experiment. When the experiment ended participants were debriefed and compensated. Each participant took part only in one of the four experiments reported here. We turn to elaborate the specific methods of each experiment.

### Exp. 1a

#### Participants

Fifty participants took part in the experiment (35 females; mean age ± SD: 23 ± 3.2).

#### Task, stimuli and design

The main memory task in this study comprised three stages: learning, validation and test stages. During the learning stage, participants viewed word pairs and were asked to associate each pair for a following recognition test. Word pairs were either previously unrelated (i.e., forming a STM association, e.g., Bottle-Phone) or previously associated in LTM (e.g., String-Guitar). All words were Hebrew nouns, taken from the Hebrew word-association norms^43^, and were controlled for length, frequency, and LTM associativity. Words were presented side by side in the middle of the screen for three seconds. Fifty-four word pairs appeared at this stage. Each pair was presented twice, and the order of presentation was randomized. At the validation stage, participants were presented again with word pairs and were asked to indicate by keypress on each trial whether the word pair appearing on the screen was learned on the first stage or not. Validation comprised 108 trials, half of which were word pairs that were presented during the learning stage. At this stage, word pairs appeared on screen until response and for the maximum of five seconds. Finally, during the test stage, participants were presented in each trial with a cue word followed by two words, a target and a distractor, presented side by side (presentation side counterbalanced). Participants were asked to indicate which of the two possibilities was associated with the cue during learning, by using the right or left arrows. Words were presented until response, and for the maximum of five seconds. The test stage involved three test conditions. In the first condition, the target was associated with the cue in LTM and the distractor was unrelated to the cue (i.e., LTM/unrelated). In the second condition, the target formed a STM association with the cue and the distractor was unrelated to the cue (i.e., STM/unrelated). In the third condition, the correct target formed a STM association with the cue and the distractor was associated with the cue in LTM (i.e., STM/LTM).

The test phase comprised eleven mini-blocks and was conducted under high or low load, depending on the group (see "General methods"). At the beginning of each mini-block, a digit-string was presented on screen for three seconds, allowing participants to memorize the digits. Digit presentation was followed by five recognition test trials, after which participants were asked to type the digits that they were asked to memorize. The last mini-block of the test stage comprised four trials of the recognition task. Altogether this experimental stage comprised a total of eleven digit-strings to remember, and fifty-four recognition trials. This experiment extended approximately 30 min.

### Exp. 1b

#### Participants

Forty-six participants took part in the experiment (29 females; mean age ± SD: 25 ± 5.1).

#### Task, stimuli and design

The experiment was identical to Exp. 1a with the only difference that participants completed two consecutive blocks of the experimental procedure instead of one, each block using a completely different set of stimuli. In the two blocks together, this experiment comprised a total of twenty-two mini-blocks of digit-strings to remember, and one hundred and eight memory recognition test trials. Between blocks, participants were given a short break and were asked to continue to the second block by using a keypress. This experiment extended approximately one hour.

### Exp. 2

#### Participants

Fifty participants took part in the experiment (32 females; mean age ± SD: 22 ± 2.7).

#### Task, stimuli and design

Exp. 2 comprised two stages- learning and test. During the learning stage, differently colored words appeared consecutively on the screen, each presented for 750 ms and followed by a 250 ms presentation of a fixation cross. Subjects were asked to associate every two consecutive words (cue-target) that appeared in the same color. Sixty word-pairs were presented in the learning stage. Each associated word-pair appeared four times throughout the learning stage, and was always preceded with the same word, which was different in color, forming an incidental association with the word-pair’s cue word. Additionally, buffer words, random in their order and color were implemented during learning as well, also appearing four times throughout the learning stage. At the test stage, participants were presented with the word-pair’s cue word and were asked to indicate which of two additional words was the word that they intentionally associated with the cue, using the right or left arrows. As there was always a correct answer, this stage differentiated between two possible conditions. In the first condition, the distractor was one of the buffer words presented during learning, and in the second condition, the distractor formed an incidental association with the cue. The cue, as well as the target and distractor words, appeared on the screen until response and for the maximum of five seconds. Similarly to Exp. 1, this test stage was conducted under low or high cognitive load, as a between-subjects variable. The test stage comprised six mini-blocks of the cognitive load task, each of which comprised ten trials of the recognition test. Each mini-block began with a 3-s presentation of a digit-string to remember. This was followed by trials of the recognition test, after which participants were asked to type the digit string they kept in mind. This experiment extended approximately 30 min.

### Exp. 3

#### Participants

Fifty participants took part in the experiment (37 females; mean age ± SD: 24 ± 4.6).

#### Task, stimuli and design

The design of Exp. 3 was similar to Exp. 2. Nonetheless, while Exp. 2 manipulated words as stimuli, Exp. 3 manipulated images. Images were taken from Brady et al.^44^.

Exp. 3 comprised a learning stage and a test stage. During the learning stage, images and image-pairs appeared consecutively on the screen, each presented for 750 ms and followed by a 250 ms presentation of a fixation cross. Given the pictorial nature of the task, color cueing for associative learning was not ideal to use. Therefore, participants were explicitly asked to associate images that were presented together, for a following recognition test. Each image pair was consistently preceded with the same image, forming a temporal, incidental association with the image pair. Buffer images, random in their presentation order were implemented as well. All images (i.e., buffer images, incidentally associated images, and image-pairs), appeared four times throughout the learning stage. A total of 60 image pairs were presented during learning. During the test stage, participants were presented with one image from each learned associated image-pair, and a side-by-side presentation of two additional images. Participants were asked to indicate which of the two images was associated with the cue during learning, by using the right or left arrows. Images were present on screen until response and for the maximum of five seconds. As in Exp. 2, this stage differentiated between two recognition conditions. In the first condition, the distractor was a buffer image that was presented during learning but was unrelated to the image-pair. In the second condition, the distractor formed an incidental association with the image-pair. Similarly to Exp. 2, the test stage was conducted under low or high cognitive load, as a between-subjects variable. Each mini-block began with a 3 s presentation of the digits to remember, followed by trials of the recognition test, after which participants were asked to type the digit-string that they memorized. The test stage comprised six mini-blocks, each of them consisting of ten recognition test trials. This experiment extended approximately 30 min.

### Exp. 4

Fifty-two participants took part in the experiment (30 females; mean age ± SD: 26 ± 4.1). One participant was excluded from analysis due to outlier performance in the load manipulation (below 2.5 std from the mean).

The primary task in Exp. 4 did not involve learning but rather employed a contextual priming paradigm that focused on object recognition. Each trial in the paradigm began with a 250 ms presentation of a prime. This was followed by a 100 ms presentation of a mask, and concluded with a presentation of the target, that was present on screen until response and for the maximum of three seconds. Half of the target images depicted real objects, and half depicted abstract, non-object sculptures. Participants were instructed to respond to the target and determine by keypress whether it depicted a real object or a non-object. With regards to real object targets, the preceding prime images could be either unrelated or associated with the target. To differentiate levels of prime-target associativity, we created three groups of prime-target associations, such that primes could be associated to the targets in one of three strength levels: strong, intermediate and weak (see Supplementary Bfor further details on stimuli selection and associative validation). Throughout the experiment, each image from the three priming groups (strong, intermediate and weak) repeated twice, once followed by a real object and once followed by a non-object (i.e., an image of an abstract sculpture). Equating the number of real objects and non-objects in the task was meant to avoid any response-related confounds. Among real-object target trials, half the primes were followed by the presentation of an object that is associated with the prime in LTM (in a strong, intermediate or weak manner), and half were followed by an unrelated object. The experiment comprised a total of 432 trials. Simultaneously to performing the object recognition task, participants were asked to perform the cognitive load task. Similarly to the previous experiments, participants were assigned to one of two cognitive load groups. In both load groups, the experiment comprised thirty-six mini-blocks. Each mini-block began with a 3-s presentation of the digit string to remember. This was followed by twelve trials of the object recognition task, after which participants were asked to type the digit string they memorized.

### Data analysis

Data was analyzed with custom software using Matlab version R2014a (MathWorks). Analysis procedure in all experiments was identical and began with analysis of accuracy in the cognitive load task. Participants who performed below 2.5 std from the mean were excluded from further analysis. Only trials in which memory of the digits was correct were included in following analysis (although see Supplementary Afor additional analysis schemes). Next, performance in the primary task was analyzed, i.e., memory recognition (Exp. 1–3) or object recognition (Exp. 4). This was done by conducting repeated measures ANOVA both for accuracy (i.e., hit rate) and RT, in which load was a between-subjects variable and memory recognition/object recognition conditions were a within-subjects variable. Here too, participants performing below 2.5 std from the mean in either accuracy or RT were excluded.

## Supplementary Information

Supplementary Information.

## Data Availability

Code for all data reported in study is available from the corresponding authors upon request.

## Code availability

Code for all experiments reported in study is available from the corresponding authors upon request.
