# Supplementary material for: Increased associative interference under high cognitive load
Source: Sci Rep. 2022 Feb 2;12:1766. doi: 10.1038/s41598-022-05722-w (PMC8811063; doi:10.1038/s41598-022-05722-w)
Supplement: Supplementary file 1 — Supplementary Information. [file 41598_2022_5722_MOESM1_ESM.docx]

**Supplementary Materials:**

**Increased associative interference under high cognitive load**

Shira Baror^1,2^ & Moshe Bar^1^

^1^The Gonda Multidisciplinary Brain Research Center, Bar Ilan University, Israel

^2^Neuroscience Institute, New York University School of Medicine, New York, NY 10016, USA

**Supplementary A: Comparing results in all experiments using 3 different criteria to evaluate performance under high load.**

**
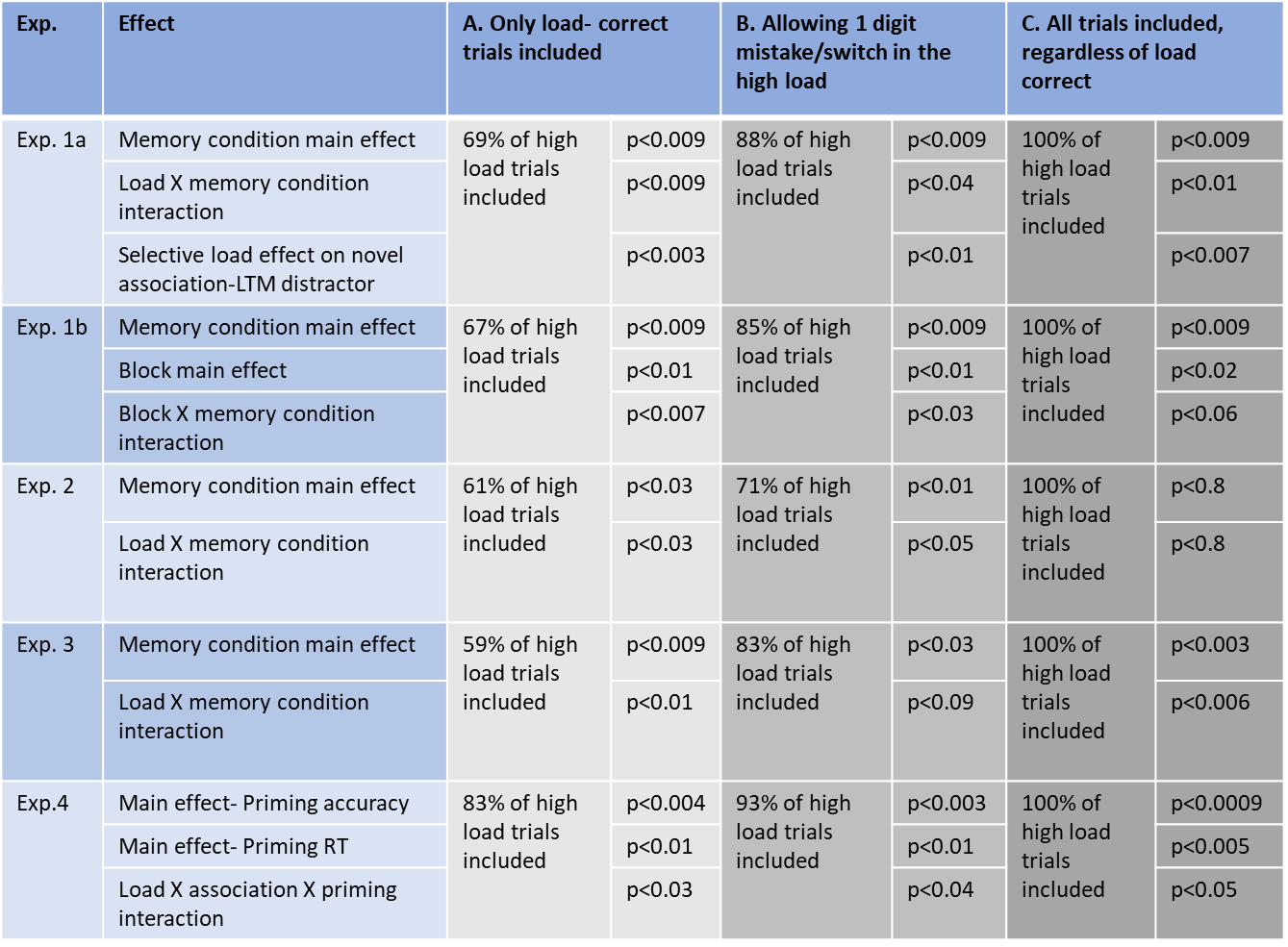
**

**Table S1:** The table depicts p values of the main effects reported in the article for the main task in all four experiments, under three different trial-inclusion criteria: **(A) Only load-correct trials included:** memory performance in the main tasks in each experiment is calculated based on trials in which load performance was 100% correct. This stringent criterion, which is the one reported in the article, is used to exclude trials in which participants may have not been occupied with the cognitive load task. While noise is minimized in this analysis, an imbalance between the number of trials included in the low and the high load conditions exists. **(B) Allowing 1 digit mistake or switch in the high load group:** memory performance in the main tasks is calculated based on trials in which load performance in the high load group was robustly but not completely correct. This analysis includes trials in which at least five digits out of the six digits to remember in the high load group were correctly remembered. While this analysis introduces some load-incorrect trials, it reduces the imbalance between the number of trials included in the low load and high load groups. **(C)** **All trials included,** **regardless of load-correct:** memory performance in the main tasks is calculated based on all trials, regardless of load performance. This lenient criterion solves the challenge of balancing the number of trials included from each load group. Nonetheless, it introduces a significant amount of noise, as it cannot be verified that participants were engaged in the cognitive load task in trials in which they are utterly wrong. As can be seen from the table, most results (except for one analysis in Exp. 2) remain significant, regardless of number of trials included. This helps confirming that a difference between the low and high load groups in the number of trials included in the analysis does not account for the results.

**Supplementary B:**

**Preparation of Strong, Intermediate and Weak image primes for Exp. 4.**

In this pilot study we construct the image prime-target associations for Exp. 4, that are divided to strong, intermediate and weak groups, based on two evaluation criteria: associative specificity and contextual specificity, two factors that are reasoned to influence associative processing.

**Method**

20 Participants were shown 480 images of objects and were asked to respond as fast as possible with the first association that came to mind in response to each image. Participants were instructed to indicate their first visual association, and refrain from using emotional or abstract descriptions. Images appeared on the screen until an association was provided, and for the maximum of ten seconds.

***Data collection and Data analysis***

The experiment and data collection were conducted by using the MATLAB software, and data analysis was conducted using SPSS software.

**Results**

20 participants took part in the pilot in exchange for course credits and have referred to all images with their first association that came to mind. 43 images were excluded from further analysis due to being provided with abstract or general associations rather than with concrete ones (e.g., Elephant-Thailand). All additional analyses include only the remaining 437 images.

Mean reaction time (RT) for responding in the task was 3.08s (SD = .59s), measured as the time from the onset of image presentation to the first letter press in typing the association.

Associative specificity was calculated as the percent of dominant answer repetitions (e.g., repetition of the word ‘fish’ in response to an image of an aquarium, across participants). This yielded a continuum of strength-by-associative specificity, ranging from 5-100%. Measured RT revealed a significant correlation between RT and associative specificity, such that the stronger the association (namely, the more specific), the faster the response (r= -.91, p< .0009, Fig. S1).


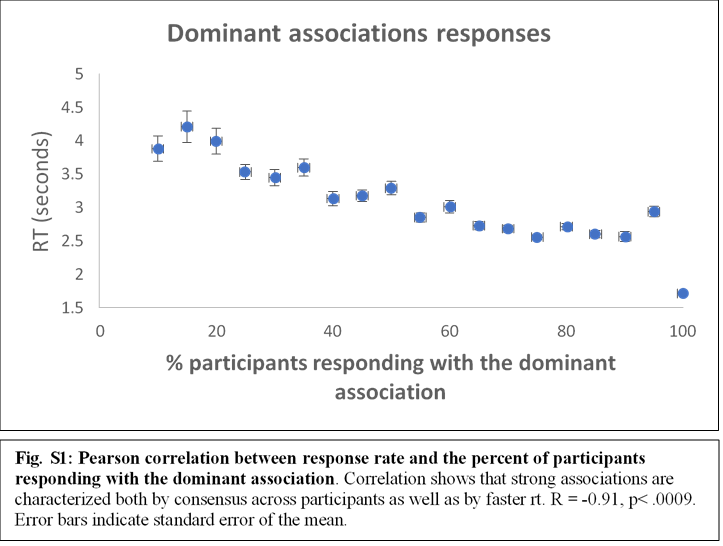


Next, every image was tagged with a binary contextual specificity index. Two raters separately evaluated whether the associations provided to it across participants belonged to a unified context or not. Objects were defined as contextually specific if more than 85% of associations given in response to them belonged to the same context.

Results in calculating associative specificity and contextual specificity yielded three groups of images:

The first group, containing 122 stimuli, comprised images that elicit one dominant association belonging to a specific context (e.g., the image of a rubber duck triggered the answer ‘bathtub’ in 95% of the responses). This group is further termed ‘strong’. The second group contained 105 stimuli and included images that elicit many associations, such that the most dominant answer was not repeated more than 35% in responses (25% on average). Nonetheless, these images triggered a specific context (e.g., the image of a parasol triggered various answers such as: ‘beach, sunglasses, towel, sand, ice cream’, all sharing the context of a beach). This group was termed ‘intermediate’. The third group contained 134 stimuli. In this group, images elicited many associations, such that the dominant answer was repeated in less than 35% of responses (21% on average). At the same time, these images were judged as contextually unspecific, such that their associations didn’t share a common context (e.g., the image of an apple triggered various answers of various contexts such as: ‘refrigerator, honey, worm, banana, tree’). This group was termed ‘weak’

The remaining images that elicited a dominant answer of between 35-70% of responses, as well as the images on which raters did not agree with regards to their contextual strength were excluded from further analysis (a total of 76 stimuli).

Further confirmatory analysis revealed that image groups differed from one another in RT, such that as expected, fastest RT was found for responding to images in the strong group (M = 2.69s SD = 1.74s). Next was RT for responding to the intermediate group of items (M = 3.53s SD =2.48s). Longest RT was found for associations given to the weak group of stimuli (M = 4.01s SD =3.08s; repeated measured ANOVA, F(57,2) = 7.45, p< .001). Table S2 summarizes the image group information from the pilot study.


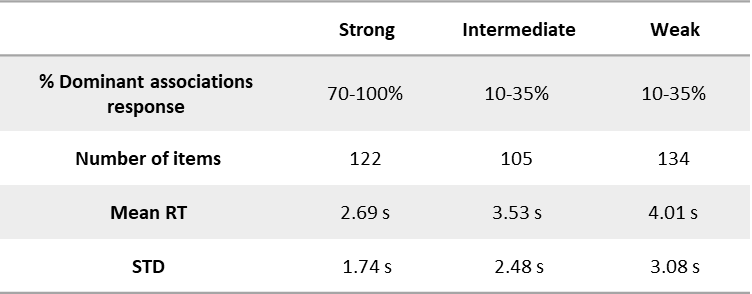


**Table S2:** Summary of associative group information. Three object groups were generated from responses to images in a free associations test. Strong: objects that trigger few dominant associations belonging to one context. Intermediate: objects that trigger many different associations that share one context. Weak: objects that trigger many different associations that do not belong to the same context.

Images from all three association groups were used as stimuli for Exp. 4.
